# Predictive factors of viral load high-risk events for virological failure in HIV/AIDS patients receiving long-term antiviral therapy

**DOI:** 10.1186/s12879-021-06162-z

**Published:** 2021-05-18

**Authors:** Shanfang Qin, Jingzhen Lai, Hong Zhang, Di Wei, Qing Lv, Xue Pan, Lihua Huang, Ke Lan, Zhihao Meng, Hao Liang, Chuanyi Ning

**Affiliations:** 1 Guangxi AIDS Diagnosis and Treatment Quality Control Center, Longtan Hospital of Guangxi Zhuang Autonomous Region, Liuzhou, 545005 Guangxi China; 2 Guangxi Key Laboratory of AIDS Prevention and Treatment, Guangxi Medical University, Nanning, 530021 Guangxi China; 3 Guangxi Collaborative Innovation Center for Biomedicine, School of Public Health & Life Sciences Institute, Guangxi Medical University, No.22 Shuangyong Road, Nanning, 530021 Guangxi China; 4 Department of Infectious Diseases, Longtan Hospital of Guangxi Zhuang Autonomous Region, No. 8 Yangjiaoshan Road, Liuzhou, 545005 Guangxi China; 5 Clinical Laboratory, Longtan Hospital of Guangxi Zhuang Autonomous Region, Liuzhou, 545005 Guangxi China; 6 Nursing College, Guangxi Medical University, No.22 Shuangyong Road, Nanning, 530021 Guangxi China

**Keywords:** HIV, AIDS, ART, Virological failure, Viral load

## Abstract

**Background:**

In the era of anti-retroviral therapy (ART), the plasma HIV viral load (VL) is an important primary indicator for monitoring the HIV treatment response. To optimize the clinical management of HIV/AIDS patients, we investigated VL high-risk events related to virological failure (VF) and further explored the preventive factors of VL high-risk events.

**Methods:**

The data were derived from China’s HIV/AIDS Comprehensive Response Information Management System. HIV infected patients who initiated or received ART in Guangxi between 2003 and 2019 were included. The contributions of VL after 6 months of ART to VF and AIDS-related death were analysed by Kaplan-Meier curves, log-rank tests and Cox regression analyses. Both descriptive analyses and bivariate logistic regression were employed to further explore the preventive factors related to VL high-risk events of VF.

**Results:**

The cumulative rates of VF in the high low-level viremia group (high LLV) (*χ*^*2*^ = 18.45; *P* <  0.001) and non-suppressed group (*χ*^*2*^ = 82.99; *P* <  0.001) were significantly higher than those in the viral suppression (VS) group. Therefore, the VL high-risk events of VF was defined as highest VL > 200 copies/ml after 6 months of ART. Compared with the VS group, the adjusted hazard risk was 7.221 (*95% CI*: 2.668; 19.547) in the high LLV group and 8.351 (*95% CI*: 4.253; 16.398) in the non-suppressed group. Compared with single patients, married or cohabiting (*AOR* = 0.591; *95% CI*: 0.408, 0.856) and divorced or separated (*AOR* = 0.425, *95% CI*: 0.207, 0.873) patients were negatively associated with VL high-risk events. So were patients acquired HIV homosexually (*AOR* = 0.572; *95% CI*: 0.335, 0.978). However, patients who had ART modification were 1.728 times (*95% CI*: 1.093, 2.732) more likely to have VL high-risk events, and patients who used cotrimoxazole during ART were 1.843 times (*95% CI*: 1.271, 2.672) more likely to have VL high-risk events.

**Conclusions:**

A VL greater than 200 copies/ml is a VL high-risk event for VF. Intervention measurements should be adopted to optimize the surveillance of ART in patients who are single or widowed, who have ART modification, and who use cotrimoxazole during ART.

## Background

Human immunodeficiency virus (HIV) infection and acquired immunodeficiency syndrome (AIDS) are threats to public health worldwide. The World Health Organization (WHO) estimated that 38 million individuals worldwide were living with HIV by the end of 2019 [1], and nearly 1.7 million of them were new HIV infections [2]. Fortunately, antiretroviral therapy (ART) is applied to treat HIV-1 infection and improve the life expectancy of HIV/AIDS patients. To mark the roadmap to the elimination of the AIDS epidemic as a public health threat by 2030 [3], the Joint United Nations Programme on HIV and AIDS (UNAIDS) set the global targets to have 90% of all HIV-infected individuals being diagnosed, 90% of those diagnosed being on ART and 90% of those on ART achieving viral suppression by 2020 (90–90-90 goal). However, only 67% of individuals living with HIV received ART by the end of 2019, as estimated by the WHO [4]. At the beginning of 2021, the 90–90-90 goal has fallen short.

Since the first ART drug was approved, the survival of ART-treated individuals living with HIV has improved because the ART regimens developed reduced HIV viral loads and delayed disease progression. Combination ART was developed to substantially arrest viral replication by targeting and interrupting different stages of the virus life cycle [5, 6]. As recommended by WHO, ART should be administered to all individuals as early as possible following the HIV diagnosis. The rapid popularisation of ART to treat HIV infection in low- and middle-income countries has successfully saved millions of lives [7, 8].

Adhering to the ART regimen is crucial to achieve and maintain viral suppression to prevent further HIV transmission and the progression of HIV infection [9, 10]. Furthermore, adherence to ART over 95% of the time is required to achieve full viral load suppression. When patients fail to attain the required adherence level, they may be subjected to a poorer prognosis, higher morbidity and mortality, and the development of resistance to ART [11, 12]. However, adherence to ART can only be measured by patients’ self-report, rather than an objective index. Therefore, monitoring adherence to ART for ART-experienced patients is challenging.

In clinical practice, the plasma HIV viral load (VL) is an important and primary indicator to monitor the HIV treatment response. Additionally, VL can indicate adherence to ART by showing the therapeutic response. According to the China National Handbook on Free Anti-retroviral Therapy for AIDS (4th edition), two consecutive confirmed viral loads > 400 copies/ml after 24 weeks of ART could be defined as virological failure (VF) [13]. However, there are different definitions of VF. According to the Department of Health and Human Services (DHHS, USA) guidelines, a confirmed viral load > 200 copies/ml can be defined as virological failure (VF) [14]. Viral suppression (VS) is achieved if the plasma HIV-1 VL cannot be detected by routine assays (< 20–50 copies/ml). There is a “grey zone” between the VF and VS, usually known as low-level viremia (LLV). Persistent LLV may contribute to an increased risk of VF. A large study in South Africa reported that LLV is not only a risk factor for VF but also decreases the durability of viral re-suppression [15]. Additionally, drug resistance, immunological failure, non-AIDS comorbidities, clinical progression to AIDS and AIDS-related death were correlated with LLV [16]. Therefore, LLV is likely a risk factor resulting in adverse clinical outcomes for HIV/AIDS patients. The occurrence of LLV should be prevented if possible. However, no general agreement exists regarding a standard definition of LLV.

To optimize the clinical management of HIV/AIDS patients, we investigated the risk events related to VF and AIDS-related death based on the data of patients who initiated or received ART in Guangxi between 2003 and 2019 exported from China’s HIV/AIDS Comprehensive Response Information Management System (CRIMS). Furthermore, to prevent VL risk events as early as possible and block the occurrence of subsequent adverse events during ART, we explored the preventive factors of risk events.

## Methods

### Study design and population

**Fig. 1:**
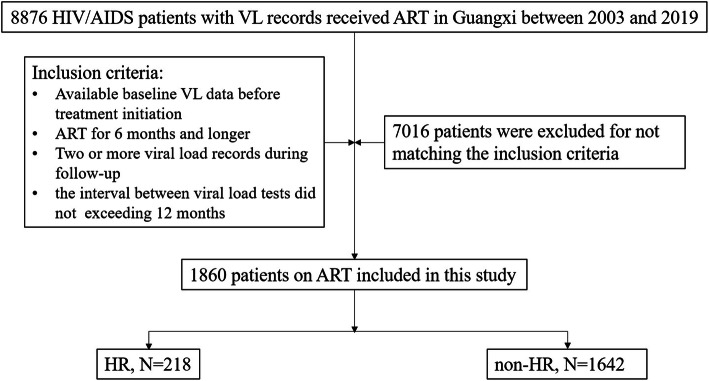
Flowchart of the patients’ inclusion criteria. VL: viral load. HR includes patients who experienced VL high-risk events of VF, while non-HR includes patients who did not experienced VL high-risk events of VF

A retrospective follow-up study was conducted among HIV/AIDS patients who initiated or received ART in Guangxi, China between 2003 and 2019. The follow up period was the time between ART initiation and a certain outcome was confirmed. We utilized data from China’s HIV/AIDS CRIMS. The patients’ inclusion criteria were as follows: a) available baseline VL data before treatment initiation; b) ART for 6 months and longer; c) two or more viral load records during follow-up; and d) the interval between viral load tests not exceeding 12 months. Figure 1 shows a flowchart of the patients’ inclusion criteria.

### Definitions and data collection

Demographic information was collected from the baseline of ART initiation, such as sex, marital status, age at HIV diagnosis, age at ART initiation and registered permanent residence. At ART initiation, HIV infection-related information was also collected, such as the transmission route, *Mycobacterium tuberculosis* (Mtb) infection in the recent year, opportunistic infections (OIs) in the recent 3 months, AIDS-related symptoms, WHO clinical stage, and VL. VL, duration on ART (months), ART regimen and ART regimen switch or interruption during the ART follow-up period were collected.

Based on the 4th edition of the China National Handbook of Free Antiviral Therapy for AIDS, VF was defined as a viral load greater than 400 copies/ml two or more consecutive times during ART, whereas VS was defined as a VL below the minimum detection threshold of the detection reagent (usually below 20 copies/ml). VLs between 20 copies/ml and 400 copies/ml were divided into two subgroups: low LLV (20 copies/ml ≤ VL ≤ 200 copies/ml) and high LLV (200 copies/ml < VL ≤ 400 copies/ml). Therefore, the outcome of VF was defined as VL > 400 copies/ml two or more consecutive times after 6 months of ART. The VLs after 6 months of ART were records of VL detected between 6 months after ART and the time of first VL > 400 copies/ml of VF or death or the last VL detection. VLs after 6 months of ART were divided into four groups, including VS (all VLs < 20 copies/ml); low LLV (20 copies/ml ≤ VL ≤ 200 copies/ml); high LLV (200 copies/ml < VL ≤ 400 copies/ml) and non-suppressed (VL > 400 copies/ml).

### Statistical analysis

To determine the contribution of VL after 6 months of ART to VF and AIDS-related death, the cumulative rates of VF and AIDS-related death were evaluated by Kaplan-Meier curves and tested by the log-rank test. Cox regression analyses were performed to investigate whether different VLs were associated with VF. Hazard ratios were adjusted for demographic information, HIV infection-related information and ART follow-up data. Based on the results of analysis above, we define that a VL greater than 200 copies/ml after 6 months of ART (high LLV group and non-suppressed group) as VL high-risk event for VF. To further explore the preventive factors related to VL high-risk events of VF, patients after antiviral therapy were divided into two groups: patients who experienced VL high-risk events of VF (HR) and those who did not (non-HR). Demographic information, HIV infection-related information and ART follow-up data were compared between these groups by *chi-square* test or the *Mann-Whitney U* test. Variables significantly different between the groups were further entered into a bivariate logistic regression model to identify independent factors correlated with LLV occurrence. All statistical analyses were performed using IBM SPSS statistics 23.0 (IBM Corp, Armonk, NY, USA). All the tests were two-sided, and *P* <  0.05 was considered to be significant.

## Results

### Incidence of VF and AIDS-related death

We recruited 1860 HIV-infected patients based on the inclusion criteria. They were followed up for a total of 6455.34 person years. After 6 months of ART, VF and AIDS-related death were set as the outcome indicators when following HIV patients. Additionally, the VL between 6-month ART and VF was recorded. Patients were divided into four groups based on the recorded VL, including the viral suppression group (VS; VL < 20 copies/ml), low LLV group (20 copies/ml ≤ VL ≤ 200 copies/ml), high LLV group (200 copies/ml < VL ≤ 400 copies/ml), and non-suppressed group (VL > 400 copies/ml).

**Fig. 2:**
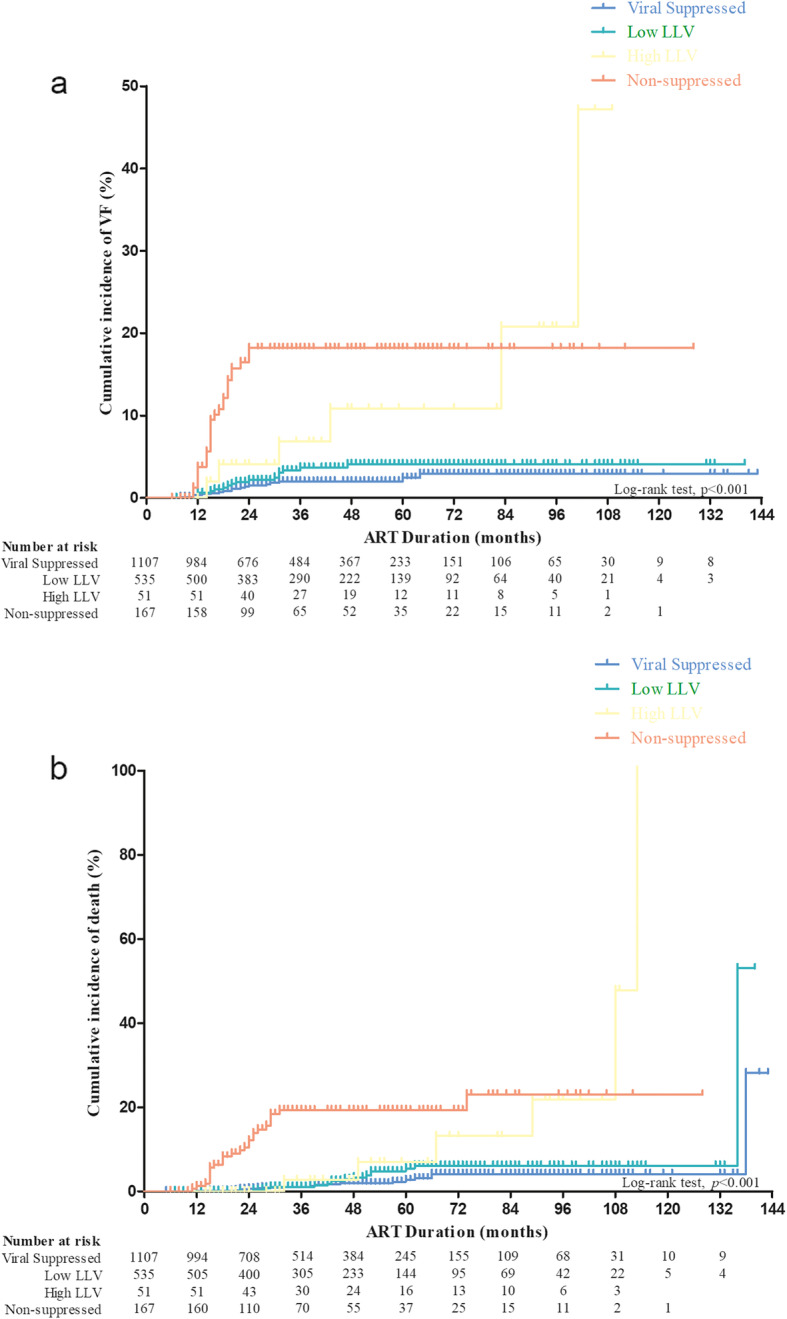
Cumulative incidence of viral failure (VF) and AIDS-related death in the different groups. **a** Cumulative incidence of VF among different groups, and **b** cumulative incidence of AIDS-related death among different groups. Patients were divided into four groups: viral suppressed (all VLs < 20 copies/ml), low LLV (20 copies/ml ≤ VL ≤ 200 copies/ml), high LLV (200 copies/ml < VL ≤ 400 copies/ml) and non-suppressed (VL > 400 copies/ml), based on the viral loads detected between 6 months after ART initiation and a certain outcome. Number at risk represents the number of patients who did not have VF and AIDS-related death, over different duration of ART in these four groups

The cumulative rates of VF and AIDS-related death were calculated by *Kaplan-Meier* analysis and were further compared among groups by *log-rank tests*. The cumulative incidence rates of VF and AIDS-related death were significantly different among the four groups (*P* <  0.001; Fig. 2). The difference in the cumulative rate of VF between the VS and low LLV groups was insignificant (*χ*^*2*^ = 1.921; *P* = 0.166). However, the cumulative rates of VF in the high LLV group (*χ*^*2*^ = 18.45; *P* <  0.001) and non-suppressed group (*χ*^*2*^ = 82.99; *P* <  0.001) were significantly higher than those in the VS group. The cumulative incidence of AIDS-related death was highest in the high LLV group (Fig. 2b). Furthermore, the cumulative incidence of AIDS-related death in the high LLV group was significantly higher than that in the VS group (*χ*^*2*^ = 15.51; *P* <  0.001) and low LLV group *(χ*^*2*^ = 8.061; *P* = 0.0045). However, no significant difference was found in the cumulative incidence of AIDS-related death between the high LLV and non-suppressed group (*χ*^*2*^ = 2.115; *P* = 0.1459).

Univariate and multivariate Cox regression models were used to determine the effect of VL after 6 months of ART on VF. The time variable was the interval between ART initiation to the outcome (VF or not). Compared with the VS group, the adjusted hazard risk was 7.221 (95% CI: 2.668, 19.547) in the high LLV group and 8.351 (95% CI: 4.253, 16.398) in the non-suppressed group (Table 1). In this study, we supposed that a VL greater than 200 copies/ml after 6 months of ART (high LLV group and non-suppressed group) is an independent VL high-risk event for VF.

**Table 1:** Effect of the viral load on virological failure after 6 months of ART

VL after 6 months of ART	Univariable analysis		Multivariable analysis	
	Crude HR (95% CI)	*P*	Adjusted HR (95% CI)^a^	*P*
**VS**	1	< 0.001	1	< 0.001
**Low LLV**	1.608 (0.820, 3.153)	0.167	1.516 (0.723, 3.180)	0.271
**High LLV**	5.989 (2.377, 15.091)	< 0.001	7.221 (2.668, 19.547)	0.005
**Non-suppressed**	9.717 (5.351, 17.646)	< 0.001	8.351 (4.253, 16.398)	< 0.001

^a^HR was adjusted by sex, marital status, age when HIV infection was confirmed, age at ART initiation, transmission route, TB infection in the recent year at ART initiation, opportunistic infection in the last 3 months at ART initiation, HIV symptoms, clinical stage, CD4+ T-cell count, VL at baseline, time between HIV diagnosis and ART initiation, ART regimen at initiation, ART regimen switch, times of ART regimen switch, ART interruption and cotrimoxazole usage before and during ART

To prevent VF at an early stage, the independent risk factors for patients who had a VL greater than 200 copies/ml after 6 months of ART were explored. Patients included in this study were divided into a high-risk group and a non-high-risk group based on VL after 6 months of ART. The high-risk group (HR) comprised patients who had a VL greater than 200 copies/ml after 6 months of ART, while the non-high-risk group (non-HR) comprised patients whose VL was not greater than 200 copies/ml after 6 months of ART.

### Demographic characteristics of the high-risk group

According to the sociodemographic characteristics of the patients at baseline, most were male (70.70%) and married or cohabiting (56.88%), with a median age at the HIV diagnosis of 39 years and a median age at ART initiation of 40 years. Sex and age at HIV diagnosis and ART initiation were not significantly different between the HR and non-HR groups. However, compared with the non-HR group, the marital status of the HR group was significantly different (*χ*^*2*^ = 10.812; *P* = 0.029; Table 2).

**Table 2:** Baseline demographic characteristics of ART-treated HIV/AIDS patients in Guangxi between 2003 and 2019

Variables	Total (%) (*n* = 1860)	HR (%) (*n* = 218)	non-HR (%) (*n* = 1642)	*χ*^*2*^*/Z*	*P*
**Sex**				1.556	0.212
**Male**	1315 (70.70)	162 (74.31)	1153 (70.22)		
**Female**	545 (29.30)	56 (25.69)	489 (29.78)		
**Marital status**				10.812	0.029
**Single**	532 (28.60)	68 (31.19)	464 (28.26)		
**Married or cohabiting**	1058 (56.88)	112 (51.38)	946 (57.61)		
**Divorced or separated**	132 (7.10)	12 (5.50)	120 (7.31)		
**Widowed**	132 (7.10)	24 (11.01)	108 (6.58)		
**Unknown**	6 (0.32)	2 (0.92)	4 (0.24)		
**Age at HIV diagnosis (years) (Median, IQR)**	39 (29, 53)	40 (29.75, 55.25)	39 (29, 52.25)	−1.014	0.311
**Age at ART initiation (years) (Median, IQR)**	40 (30, 53)	41 (30, 56)	40 (30, 53)	−1.066	0.286
**Total**	1860 (100.00)	218 (11.72)	1642 (88.28)		

### HIV infection characteristics of the high-risk group

Most of the included patients were infected by HIV heterosexually. However, the HIV transmission routes of patients in the HR group were significantly different from those in the non-HR group (*χ*^*2*^ = 10.158; *P* = 0.038). The rate of OIs in the last 3 months in the HR group was significantly higher than that in the non-HR group (43.12% vs 30.02%; χ^2^ = 15.511; *P* <  0.001). Most of the patients were WHO stage I (Table 3). However, a significant difference was found in the WHO clinical stage between the HR and non-HR groups (*P* = 0.015). Additionally, the CD4^+^ T-cell count of the HR group was significantly lower than that of the non-HR group (*P* <  0.001), while the VL at baseline of the HR group was much higher than that of the non-HR group (*P* = 0.001). However, no significant difference was found in the rates of Mtb infection in recent years, positive AIDS-related symptoms or cotrimoxazole use before baseline between the HR and non-HR groups.

**Table 3:** Characteristics of HIV infection of HIV/AIDS patients treated with ART in Guangxi between 2003 and 2019

Variables	Total (%) (n = 1860)	HR (%) (n = 218)	non-HR (%) (n = 1642)	*χ*^*2*^*/Z*	*P*
**Transmission route**				10.158	0.038
**Heterosexual**	1438 (77.31)	176 (80.73)	1262 (76.86)		
**Intravenous drug use or blood exchange**	53 (2.85)	11 (5.05)	42 (2.56)		
**Homosexual**	308 (16.56)	24 (11.01)	284 (17.30)		
**Vertical**	7 (0.38)	0 (0.00)	7 (0.43)		
**Unknown**	54 (2.90)	7 (3.21)	47 (2.86)		
**Mtb infection in the recent year**				5.007	0.082
**Yes**	164 (8.82)	28 (12.84)	136 (8.28)		
**No**	1680 (90.32)	188 (86.24)	1492 (90.86)		
**Unknown**	16 (0.86)	2 (0.92)	14 (0.85)		
**OIs in the recent 3 months**				15.511	< 0.001
**Yes**	587 (31.56)	94 (43.12)	493 (30.02)		
**No**	1244 (66.88)	122 (55.96)	1122 (68.33)		
**Unknown**	29 (1.56)	2 (0.92)	27 (1.64)		
**AIDS-related symptoms**				0.532	0.466
**Yes**	252 (13.55)	33 (15.14)	219 (13.34)		
**No**	1608 (86.45)	185 (84.86)	1423 (86.66)		
**WHO clinical stage**				10.499	0.015
**I**	1068 (57.42)	103 (47.25)	965 (58.77)		
**II**	202 (10.86)	29 (13.30)	173 (10.54)		
**III**	218 (11.72)	31 (14.22)	187 (11.39)		
**IV**	372 (20.00)	55 (25.23)	317 (19.31)		
**CD4**^**+**^ **T-cell count (cells/ml)**	211 (58, 329)	156 (33, 294)	220 (62.5, 333)	−3.604	< 0.001
**Viral Load (Log10 copies/ml) (Median, IQR)**	4.89 (4.37, 5.38)	5.08 (4.38, 5.60)	4.85 (4.31, 5.35)	−3.467	0.001
**Cotrimoxazole use before baseline**				1.499	0.221
**Yes**	573 (30.81)	75 (34.40)	498 (30.33)		
**No**	1287 (69.19)	143 (65.60)	1144 (69.67)		
**Total**	1860 (100.00)	218 (11.72)	1642 (88.28)		

### Characteristics of ART in the high-risk group

The median time between HIV diagnosis and ART initiation was 18 days in all patients included in this study. Most were treated with the NRTI + NNRTI regimen. The time between the HIV diagnosis and ART initiation and the ART regimen were not significantly different. However, the rate of ART modification was much higher in the HR group (12.84% vs 7.73%; *χ*^*2*^ = 6.577; *P* = 0.010). Additionally, the percentage of cotrimoxazole use during ART in the HR group was much higher than that in the non-HR group (40.83% vs 25.40%; *χ*^*2*^ = 23.136; *P* <  0.001; Table 4).

**Table 4:** Characteristics of ART in HIV/AIDS patients treated with ART in Guangxi between 2003 and 2019

Variables	Total (%) (n = 1860)	HR (%) (n = 218)	non-HR (%) (n = 1642)	*χ*^*2*^*/Z*	*P*
**Time between HIV diagnosis and ART initiation (days) (Median, IQR)**	18 (8, 64.75)	19 (7, 71.75)	18 (8, 64)	−0.119	0.905
**ART regimen at ART initiation**				–	0.419
**NRTI**	15 (0.81)	2 (0.92)	13 (0.79)		
**NRTI + NNRTI**	1628 (87.53)	186 (85.32)	1442 (87.82)		
**NRTI + PI**	164 (8.82)	21 (9.63)	143 (8.71)		
**NRTI + INSTI**	8 (0.43)	0 (0.00)	8 (0.49)		
**NNRTI**	13 (0.70)	3 (1.38)	10 (0.61)		
**NNRTI + PI**	23 (1.24)	5 (2.29)	18 (1.10)		
**PI**	9 (0.48)	1 (0.46)	8 (0.49)		
**ART modification**				6.577	0.010
**Yes**	155 (8.33)	28 (12.84)	127 (7.73)		
**No**	1705 (91.67)	190 (87.16)	1515 (92.27)		
**Times of ART modification**				–	0.011
**0**	1705 (91.67)	190 (87.16)	1515 (92.27)		
**1**	139 (7.47)	28 (12.84)	111 (6.76)		
**2**	14 (0.75)	0 (0.00)	14 (0.85)		
**3**	2 (0.11)	0 (0.00)	2 (0.12)		
**ART interruption**				–	0.117
**Yes**	1 (0.05)	1 (0.46)	0 (0.00)		
**No**	1859 (99.95)	217 (99.54)	1642 (100.00)		
**Cotrimoxazole use during ART**				23.136	< 0.001
**Yes**	506 (27.20)	89 (40.83)	417 (25.40)		
**No**	1354 (72.80)	129 (59.17)	1225 (74.60)		
**Total**	1860 (100.00)	218 (11.72)	1642 (88.28)		

### Factors related to VL high-risk events for VF

To determine the independent factors related to VL high-risk events for VF, variables significantly different between the two groups were entered into a bivariate logistic regression model, such as the marital status, transmission route, OIs in the last 3 months, WHO clinical stage, baseline CD4^+^ T-cell count, baseline VL, ART modification and cotrimoxazole use during ART (Table 5). The occurrence of VL high-risk events for VF (a score of 1 for Yes, 0 for No) was defined as the dependent variable. Compared with single patients, married or cohabiting (*AOR* = 0.591; *95% CI*: 0.408, 0.856) and divorced or separated (*AOR* = 0.425; *95% CI*: 0.207, 0.873) patients were negatively related to VL high-risk events. Patients who acquired HIV homosexually showed a negative relationship with VL high-risk events (*AOR* = 0.572; *95% CI*: 0.335, 0.978). However, patients who had ART modification were 1.728 times (95% CI: 1.093, 2.732) more likely to have VL high-risk events, and patients who used cotrimoxazole during ART were 1.843 times (95% CI: 1.271, 2.672) more likely to have VL high-risk events.

**Table 5:** Multivariate logistic regression analysis for factors associated with virological failure related VL high-risk events

Variables	*B*	*S.E.*	*Wald*	*AOR (95% CI)*	*P value*
**Marital status**					
**Single**			17.342	1	0.002
**Married or cohabiting**	−0.527	0.189	7.733	0.591 (0.408, 0.856)	0.005
**Divorced or separated**	−0.856	0.368	5.428	0.425 (0.207, 0.873)	0.020
**Widowed**	0.117	0.280	0.175	1.124 (0.649, 1.947)	0.676
**Unknown**	1.270	0.901	1.988	3.562 (0.609, 20.820)	0.159
**Transmission route**					
**Heterosexual**			8.659	1	0.034
**Intravenous drug use or blood exchange**	0.572	0.369	2.405	1.771 (0.860, 3.647)	0.121
**Homosexual**	−0.558	0.273	4.165	0.572 (0.335, 0.978)	0.041
**Unknown**	0.380	0.423	0.806	1.462 (0.638, 3.351)	0.369
**OIs in the recent 3 months**					
**Yes**			4.407	1	0.110
**No**	−0.386	0.225	2.943	0.679 (0.437, 1.057)	0.086
**Unknown**	−1.125	0.777	2.097	0.325 (0.071, 1.488)	0.148
**WHO Clinical stage**					
**I**			1.577	1	0.665
**II**	0.138	0.268	0.264	1.148 (0.678, 1.942)	0.607
**III**	−0.036	0.285	0.016	0.964 (0.552, 1.684)	0.898
**IV**	−0.205	0.278	0.541	0.815 (0.472, 1.406)	0.462
**Baseline CD4+ T-cell count (cells/ml)**	0.000	0.001	0.141	1.000 (0.999, 1.001)	0.708
**Baseline Viral Load (Log10 copies/ml)**	0.089	0.090	0.969	1.093 (0.916, 1.305)	0.325
**ART modification**	0.547	0.234	5.482	1.728 (1.093, 2.732)	0.019
**Cotrimoxazole use during ART**	0.611	0.190	10.384	1.843 (1.271, 2.672)	0.001

## Discussion

Our study reported that HIV patients with a VL greater than 200 copies/ml were at a higher risk of VF and AIDS-related death. Among the ART-treated HIV patients, those who were married or cohabiting (*AOR* = 0.591; *95% CI*: 0.408, 0.856) or divorced or separated (*AOR* = 0.425; *95% CI*: 0.207, 0.873) and acquired HIV homosexually (*AOR* = 0.572; *95% CI*: 0.335, 0.978) were less likely to have VL high-risk events, while those who had ART modification and used cotrimoxazole during ART were 1.728 times (95% CI: 1.093, 2.732) and 1.843 times (95% CI: 1.271, 2.672) more likely to have VL high-risk events.

In this study, we reported that patients with VL reaching 200 copies/ml after 6 months of therapy were at a higher risk of VF and AIDS-related death. A retrospective cohort study indicated that among patients with persistent LLV (PLV, defined as persistent plasma viral loads of 51–1000 copies/ml for at least 3 months), a PLV > 400 copies/mL (hazard ratio = 3.3; *95% CI:* 1.5–7.1; *P* = 0.003) predicted VF [17]. Additionally, Quiros-Roldan et al [18] reported that patients with persistent LLV (37–200 copies/ml) did not have an increased risk of death compared with those with sustained VS. [19] Therefore, these two studies may support the association between VL greater than 200 copies/ml and an increased risk of VF and AIDS-related death.

Many studies have reported that respondents who were married and/or ever married were more likely to be associated with VS. [20, 21] Additionally, among individuals with a non-suppressed viral load, marriage was associated with lower odds for VF [22]. In our study, we found that married or cohabiting and divorced patients were negatively associated with VL high-risk events (VL > 200 copies/ml). Marriage ensures a stable environment and treatment support from a partner and is crucial for HIV patients [21]. Patients who are married or cohabiting show better adherence to ART. However, married patients may be more willing to reduce the chances of HIV transmission to uninfected partners; thus, they would respond to growing public health education on the importance of taking ART [23, 24]. Single patients and widowed patients may have less social support and an unstable living environment, which may affect their adherence to ART. Therefore, single patients and widowed patients may be the key population to target to prevent and control VL high-risk events for VF and AIDS-related death.

In this study, we reported that patients who acquired HIV homosexually were less likely to have VL high-risk events. Similar results have been reported previously. Factors associated with a high HIV suppression rate in ART-treated AIDS patients were infection through homosexual transmission (OR = 0.57; 95% CI: 0.35–0.90) [25]. A large cohort study in Hunan, China, reported that both cumulative immunological and virological failure rates (10.4 and 26.4% in 48 months, respectively) were the lowest in MSM compared with other population groups [26]. Additionally, Saunder et al. [27] reported that MSM were at a considerably lower risk of VF and less common treatment switches. An explanation for our findings may be that MSM showed better management in HIV/AIDS surveillance and treatment. In China, HIV/AIDS prevention measurements targeting MSM have led to increased earlier HIV testing and improved adherence behaviours [28]. As the acceptance of MSM increases [29], a supportive healthcare environment was built to ensure the accessibility of medical services to MSM infected with HIV [30, 31]. MSM were diagnosed with HIV infection earlier, started ART with less advanced disease and presented earlier for care [27]. MSM were also more likely to receive regular CD4^+^ cell count and HIV viral load monitoring, less likely to report missing ART doses, interrupt ART, or be lost to follow-up than women who have sex with men.

Patients who had ART modification were more likely to have VL high-risk events. Some cohort studies have reported high rates of change in the first ART [32–34]. ART regimens often need to be replaced because of virological failure or toxicity or for simplification [32, 35, 36]. Approximately 29.7% of patients start ART-modified regimens within the first year after ART initiation, with half of these changes being due to drug intolerance and/or toxicity [32]. In resource-limited settings, the rates of ART modification are lower and mainly due to virological failure [32–34, 37]. Therefore, ART modification may be directly due to a higher viral load, including the VL high-risk event defined in this study. Different ART regimens showed different rates of modification; for example, patients on lopinavir and other protease inhibitors had higher rates of modification than those on efavirenz [38]. After appropriate ART modification, the 6-month and long-term rates of VF and drug resistance are low [39, 40]. However, an unnecessary ART switch increases the risk of developing drug resistance and other poor outcomes, particularly switching to third-line ART regimens, which help to damage this last line of defence against HIV. In this study, the standards for ART modification should be strict, and proper follow-up should be conducted after ART modification to avoid the incidence of VL high-risk events after switching.

Cotrimoxazole, a fixed-dose combination of trimethoprim and sulfamethoxazole, is widely used against opportunistic infections in patients with advanced HIV disease [41–43]. Increasing availability of cotrimoxazole has resulted in a decline in mortality in individuals living with HIV (PLHIV) [43, 44]. In 2017, WHO published guidelines recommending cotrimoxazole prophylaxis to reduce morbidity and mortality among PLHIV whose CD4 count was ≤350 cells/mm^3^ or at clinical stage 3 or 4 or lifelong cotrimoxazole for any CD4 count in settings with a high prevalence of malaria or bacterial infections. In China, a national free ART programme was officially launched in 2003, and the first guidelines on cotrimoxazole were released in 2005 [45]. Currently, the criteria for cotrimoxazole prophylaxis among PLHIV older than 14 years in China are a CD4^+^ T-cell count less than 200 cells/μL or a total lymphocyte count less than 1200 cells/μL, WHO stage 4 disease or a history of oropharyngeal candidiasis [13]. In this study, the patients who reported cotrimoxazole utilization all had baseline CD4^+^ T-cell counts less than 200 cells/μL and/or a high VL. Therefore, patients who used cotrimoxazole during ART were more likely to have VL high-risk events in this study. To the best of our knowledge, continued cotrimoxazole prophylaxis therapy can significantly reduce the risk of severe opportunistic infection in ART patients [46]. However, discontinuing cotrimoxazole use may result in an increased incidence of malaria in a malaria-endemic region [47]. Sisay M et al. [48] reported that the wide utilization of cotrimoxazole to prevent opportunistic infection may contribute to the emergence of antimicrobial resistance. Hence, it is necessary to initiate, fine-tune and stop the utilization of cotrimoxazole.

This study has several limitations. First, because the interval between the viral load tests of all the included patients did not exceed 12 months, some patients who did not undergo viral load testing within 12 months were excluded. Thus, the representativeness of the participants was affected. Second, we did not collect the data of non-AIDS-related deaths in this study, which may be associated with VL high-risk events too. Third, some unmeasured potential confounders could also result in the difference between groups. What’s more, we did not collect data to evaluate ART adherence, which may be associated with VL high-risk events, VF and AIDS-related death. Finally, an ART drug resistance test was not performed to determine its impact on VL high-risk events.

## Conclusions

This study demonstrated that a VL greater than 200 copies/ml is a VL high-risk event for VF. To better prevent VF and AIDS-related death, intervention measures should be adopted to optimize the surveillance of ART in patients who are single or widowed, who have ART modification, and who used cotrimoxazole during ART.

## Data Availability

The datasets generated and/or analysed during the current study are not publicly available because of ethical and legal reasons but are available from the corresponding author Chuanyi Ning on reasonable request.

## Abbreviations

AIDS: acquired immunodeficiency syndrome
ART: antiretroviral therapy
CRIMS: Comprehensive Response Information Management System
DHHS: the Department of Health and Human Services
HIV: Human immunodeficiency virus
HR: high-risk group
LLV: low-level viremia
Mtb: *Mycobacterium tuberculosis*
non-HR: non-high-risk group
OIs: opportunistic infections
PLV: persistent LLV
UNAIDS: the Joint United Nations Programme on HIV and AIDS
VL: viral load
VF: virological failure
VS: viral suppression
WHO: the World Health Organization
